# Hydrogen Sulfide and Reactive Oxygen Species, Antioxidant Defense, Abiotic Stress Tolerance Mechanisms in Plants

**DOI:** 10.3390/ijms23169463

**Published:** 2022-08-21

**Authors:** Jing Zhang, Francisco J. Corpas, Jisheng Li, Yanjie Xie

**Affiliations:** 1 Laboratory Center of Life Sciences, College of Life Sciences, Nanjing Agricultural University, Nanjing 210095, China; 2 Department of Biochemistry, Cell and Molecular Biology of Plants, Estación Experimental del Zaidín, Consejo Superior de Investigaciones Científicas (CSIC), 18008 Granada, Spain; 3 College of Life Sciences, Northwest A&F University, Yangling, Xianyang 712100, China

Various stress conditions, such as drought, salt, heavy metals, and extreme temperatures, have severe deleterious effects on plant growth and directly lead to a decline in yield and quality. The exposure of plants to such abiotic stresses leads to the uncontrolled overproduction of reactive oxygen species (ROS), which are highly toxic and can impair proteins, lipids, and nucleic acids, ultimately resulting in oxidative stress. Scientists have agreed that ROS are important signaling molecules involved in the regulation of gene expression during plant growth, development, and stress responses [1]. The healthy growth of plants in response to abiotic stress is inseparable from the joint action of many metabolic regulators, among which the behaviors of signaling molecules are universal.

Hydrogen sulfide (H_2_S), which was previously considered to be toxic, has now been regarded as a burgeoning endogenous gaseous transmitter [2]. H_2_S plays a vital role in the response/adaptation mechanisms to adverse environmental conditions, as well as crosstalk with other signaling molecules, including ROS, by affecting corresponding gene expression and subsequent enzyme activities. H_2_S can provoke reversible oxidative posttranslational modification to the cysteine residues of proteins, called persulfidation, which affect the redox status and function of the target proteins. These concepts were the basis of the Special Issue “Hydrogen Sulfide and Reactive Oxygen Species, Antioxidant Defense, Abiotic Stress Tolerance Mechanisms in Plants” which aims to provide the most current findings on the function of signaling molecules, including H_2_S and ROS, in higher plants. This Special Issue contains 5 reviews and 9 original research articles. All research articles mainly concentrate on the molecular mechanism of H_2_S in plant acclimatization to abiotic stress regarding its crosstalk with other signal molecules, and each of them represents an interesting approach to this topic. The significant participation of several authors and the number of contributions testifies to the considerable interest that the topic is currently receiving in the plant science community. Here, we briefly summarize the contributions included in this Special Issue.

Chilling is the widespread environmental stress caused by drastic and rapid global climate changes, severely restricting plant growth and crop production. Cucumbers (*Cucumis sativus* L.) are typical cold-sensitive plants, and generally suffer chilling injury. Zhang et al. reported on H_2_S’s important role in improving cucumber chilling tolerance, in which they revealed the crosstalk among H_2_S, hydrogen peroxide (H_2_O_2_), and auxin and its intrinsic molecular mechanism in controlling cucumber chilling tolerance [3,4]. Using a pharmacological method, they established a causal link among the different signaling molecules and suggested that H_2_O_2_, as a downstream signal of indole-3-acetic acid (IAA), mediates H_2_S-induced chilling tolerance in cucumber seedlings [3]. Furthermore, they illustrated the molecular mechanism by which H_2_S regulates chilling stress response through the regulation of auxin signaling at the transcriptional level [4]. Transcriptome analyses were able to identify a cucumber auxin response factor (ARF) gene *CsARF5*, which was differentially expressed under H_2_S treatment. The overexpression of *CsARF5* enhanced the cold stress tolerance of cucumber. This is because ARF5 can directly activate the expression of the dehydration-responsive element-binding (DREB)/C-repeat binding factor (CBF) gene *CsDREB3*, thus regulating cucumber cold stress tolerance. The regulation of the cold stress response by the CsARF5-CsDREB3 module depended on H_2_S, as the application of H_2_S scavenger hypotaurine blocked CsARF5-mediated chilling tolerance.

H_2_S also plays important role in regulating the abscisic acid (ABA) signaling pathway. ABSCISIC ACID-INSENSITIVE 4 (ABI4) is a versatile transcription factor in ABA signaling pathways. Zhou et al. suggest the molecular link between H_2_S and ABI4 through the post-translational modifications of persulfidation during seed germination and early seedling development [5]. They demonstrated that H_2_S-mediated persulfidation plays an important role in ABI4-controlled ABA signaling in Arabidopsis. H_2_S-mediated persulfidation attenuates the ABI4 degradation during seed germination. As the ABI4 level decreased during seed germination, persulfidation-attenuated ABI4 degradation, in turn, inhibited seed germination and seedling establishment. These results indicated that H_2_S has an inhibitory effect on both the germination and postgermination growth of Arabidopsis.

Another study by Zhou et al. provided insights into the molecular mechanisms underlying H_2_S-conferred rice drought tolerance and demonstrated that H_2_S-regulated rice drought tolerance may be achieved through the persulfidation of NIA2, which is a nitrate reductase (NR) isoform responsible for the main NR activity [6]. They showed that NR activity was decreased under drought stress, along with the increase in H_2_S content. The persulfidation of NIA2 led to a decrease in total NR activity in rice. Furthermore, the drought-stress-triggered inhibition of NR activity and persulfidation of NIA2 was intensified in the rice transgenic line with the overproduction of H_2_S. In agreement with these observations, mutation of *NIA2* enhanced rice drought tolerance by activating the expression of genes encoding antioxidant enzymes and ABA-responsive genes.

The critical role of H_2_S was also suggested for plants counteracting manganese (Mn) stress. Hou et al. investigated the mechanism of H_2_S participation and alleviation of Mn stress in Arabidopsis thaliana [7]. H_2_S is involved in the alleviation of Mn-induced Arabidopsis seedling growth inhibition by reducing Mn^2+^ content, reducing ROS accumulation, and enhancing antioxidant enzyme activity. They found that the L-cysteine desulfhydrase (AtLCD) is critical for endogenous H_2_S production and further regulates Arabidopsis tolerance to Mn stress.

Interestingly, another study reported on H_2_S’s involvement in plant response to the combined stress of multiple elements [8]. Mercury (Hg) is a toxic metal, even at low levels. Se (selenium) is a beneficial micronutrient for plant growth, which, at proper concentrations, can rescue plants from the toxicity of heavy metals, including Hg. However, Se application with excess concentration shows a synergistic toxic effect with Hg. Using a specific fluorescence probe, Yang et al. found that endogenous H_2_S could be triggered as a defensive signal in response to the synergistic toxicity of Hg and Se in *Brassica rapa*. Neither Hg nor Se worked alone. The defensive role of H_2_S in response to Hg and Se treatment was evaluated by the manipulation of endogenous H_2_S levels, as the increase in endogenous H_2_S was associated with the decrease in ROS level, followed by alleviating cell death and recovering root growth. Such findings extend our knowledge of plant H_2_S in response to multiple stress conditions.

N-acetyl-5-methoxytryptamine (melatonin) was known to act as a multifunctional molecule to alleviate abiotic and biotic stresses. The review paper by Gu et al. is devoted to the effects of melatonin on the plant response to heavy metal cadmium (Cd) [9]. Melatonin activates several downstream signals, such as nitric oxide (NO), H_2_O_2_, and salicylic acid (SA), which are required for plant Cd tolerance. The author summarizes the progress in various physiological and molecular mechanisms regulated by melatonin in plants under Cd stress and discussed the complex interactions between melatonin and H_2_S in the acquisition of Cd stress tolerance. This will be of considerable interest to researchers working in the field of heavy metals, and also to those investigating the crosstalk among signaling molecules in response to abiotic stress. They also discussed the crosstalk between melatonin and ROS in plant abiotic stress responses [10]. Melatonin often cooperates with other signaling molecules, such as ROS, NO, and H_2_S. The interaction between melatonin, NO, H_2_S, and ROS orchestrates the responses to abiotic stresses via signaling networks, thus conferring plant tolerance. Gu et al. summarize the roles of melatonin in establishing redox homeostasis through the antioxidant system. They also reviewed the current progress of complex interactions between melatonin, NO, H_2_S, and ROS in plant responses to abiotic stresses and highlighted the vital role of respiratory burst oxidase homologs (RBOHs) during these processes.

Plenty of achievements have been announced regarding H_2_S working in combination with other signal molecules to adapt to environmental changes. In the review article of Wang et al., the crosstalk and regulatory mechanisms of H_2_S and other signal molecules, such as NO, ABA, calcium ion (Ca^2+^), H_2_O_2_, SA, ethylene (ETH), jasmonic acid (JA), proline (Pro), and melatonin, have been summarized within the context of plant response to abiotic stresses, including maintaining cellular redox homeostasis, exchanging metal ion transport, regulating stomatal aperture, and altering gene expression and enzyme activities [11].

The review of Khan et al. not only summarizes and discusses the current understanding of the molecular mechanisms of H_2_S-induced cellular adjustments and H_2_S involvement in various signaling pathways in plants but emphasizes the recent progress in H_2_S-mediated protein persulfidation [12]. The authors point out that more fundamental research is required to investigate the fate and regulation of endogenous H_2_S production. The direct detection of endogenous H_2_S and its potential emission is still a challenge in higher plants. By using an ion-selective microelectrode and a specific gas detector, Muñoz-Vargas et al. measured the endogenous content of H_2_S and its emissions among different plant species of agronomical and nutritional interest, including pepper fruits, broccoli, ginger, and different members of the genus Allium, such as garlic, leek, and Welsh and purple onion [13]. These results provide a good example for the accurate quantification of endogenous H_2_S production and emissions in a plant.

H_2_S plays important roles in prolonging storage life and conserving the quality attributes of horticultural products. Sun et al. reported that H_2_S’s involvement in calcium deficiency induced the development of a bitter pit on the surface of apple peels [14]. They found that the calcium content, ROS, and H_2_S production were the main differences between calcium-deficient and calcium-sufficient apple peels. Four calmodulin-like proteins (CMLs), seven AP2/ERFs, and three bHLHs transcripts were significantly differentially expressed in calcium-deficient apple peels. RT-qPCR and correlation analyses further revealed that *CML5* expression was significantly positively correlated with the expression of *ERF2/17*, *bHLH2*, and H_2_S production-related genes. Therefore, the author provides a basis for studying the molecular mechanism of postharvest quality declines in calcium-deficient apples and the potential interaction between Ca^2+^ and endogenous H_2_S.

More importantly, H_2_S plays an important role in plant development and senescence. Hu et al. demonstrated that the overexpression of tomato *LCD1* increased the endogenous H_2_S content, and delayed dark-triggered chlorophyll degradation and ROS accumulation in detached tomato leaves [15]. They found that increases in the expression of chlorophyll degradation genes *NYC1*, *PAO*, *PPH*, *SGR1*, and senescence-associated genes (*SAGs*) during senescence were attenuated by *LCD1* overexpression, whereas *lcd1* mutants showed enhanced senescence-related parameters. In the review of Li et al., the regulation of H_2_S on the root system architecture (RSA) was summarized in terms of primary root growth, lateral and adventitious root formation, root hair development, and the formation of nodules [16]. The genes involved in the regulation of the RSA by H_2_S, and the relationships with other signal pathways were also discussed. This review provides a comprehensive understanding of the role that H_2_S plays in roots during development and under abiotic stress.

In conclusion, the negative consequences for crop growth and food production that are inevitably caused by global climate changes will be a major risk in the coming decades. This explains researchers’ unprecedented interest in the fields of plant fitness, productivity, and adaptation to adverse environmental conditions. The studies in this Special Issue add valuable pieces to the puzzle regarding plant response to abiotic stress, and especially the underlying regulatory mechanisms of H_2_S and its crosstalk with other signal molecules involved in this process. We are confident that they will inspire further productive research. We wish to thank all the contributors to this Special Issue and hope that it will raise interest in, and further expand our current understanding of, plant H_2_S function. Nonetheless, the current Special Issue can only cover a few parts of H_2_S’s function in plant biology; therefore, we are pleased to release “Hydrogen Sulfide and Reactive Oxygen Species, Antioxidant Defense, Abiotic Stress Tolerance Mechanisms in Plants 2.0”, to which we continue to invite our readers to contribute.

## References

[1] Mittler R., Zandalinas S.I., Fichman Y., Van Breusegem F. (2022). Reactive Oxygen Species Signalling in Plant Stress Responses. Nat. Rev. Mol. Cell Biol..

[2] Zhang J., Zhou M.J., Zhou H., Zhao D.D., Gotor C., Romero L.C., Shen J., Ge Z.L., Zhang Z.R., Shen W.B. (2021). Hydrogen sulfide (H_2_S), a signaling molecule in plant stress responses. J. Integr. Plant Biol..

[3] Zhang X., Zhang Y., Xu C., Liu K., Bi H., Ai X. (2021). H_2_O_2_ Functions as a Downstream Signal of IAA to Mediate H_2_S-Induced Chilling Tolerance in Cucumber. Int. J. Mol. Sci..

[4] Zhang X., Fu X., Liu F., Wang Y., Bi H., Ai X. (2021). Hydrogen Sulfide Improves the Cold Stress Resistance through the CsARF5-CsDREB3 Module in Cucumber. Int. J. Mol. Sci..

[5] Zhou M., Zhang J., Zhou H., Zhao D., Duan T., Wang S., Yuan X., Xie Y. (2022). Hydrogen Sulfide-Linked Persulfidation Maintains Protein Stability of ABSCISIC ACID-INSENSITIVE 4 and Delays Seed Germination. Int. J. Mol. Sci..

[6] Zhou H., Zhou Y., Zhang F., Guan W., Su Y., Yuan X., Xie Y. (2021). Persulfidation of Nitrate Reductase 2 Is Involved in L-Cysteine Desulfhydrase-Regulated Rice Drought Tolerance. Int. J. Mol. Sci..

[7] Hou L., Wang Z., Gong G., Zhu Y., Ye Q., Lu S., Liu X. (2022). Hydrogen Sulfide Alleviates Manganese Stress in Arabidopsis. Int. J. Mol. Sci..

[8] Yang L., Yang H., Bian Z., Lu H., Zhang L., Chen J. (2022). The Defensive Role of Endogenous H_2_S in *Brassica rapa* against Mercury-Selenium Combined Stress. Int. J. Mol. Sci..

[9] Gu Q., Wang C., Xiao Q., Chen Z., Han Y. (2021). Melatonin Confers Plant Cadmium Tolerance: An Update. Int. J. Mol. Sci..

[10] Gu Q., Xiao Q., Chen Z., Han Y. (2022). Crosstalk between Melatonin and Reactive Oxygen Species in Plant Abiotic Stress Responses: An Update. Int. J. Mol. Sci..

[11] Wang C., Deng Y., Liu Z., Liao W. (2021). Hydrogen Sulfide in Plants: Crosstalk with Other Signal Molecules in Response to Abiotic Stresses. Int. J. Mol. Sci..

[12] Khan M.S.S., Islam F., Ye Y., Ashline M., Wang D., Zhao B., Fu Z.Q., Chen J. (2022). The Interplay between Hydrogen Sulfide and Phytohormone Signaling Pathways under Challenging Environments. Int. J. Mol. Sci..

[13] Muñoz-Vargas M.A., González-Gordo S., Palma J.M., Corpas F.J. (2022). H_2_S in Horticultural Plants: Endogenous Detection by an Electrochemical Sensor, Emission by a Gas Detector, and Its Correlation with L-Cysteine Desulfhydrase (LCD) Activity. Int. J. Mol. Sci..

[14] Sun H.-Y., Zhang W.-W., Qu H.-Y., Gou S.-S., Li L.-X., Song H.-H., Yang H.-Q., Li W.-J., Zhang H., Hu K.-D. (2021). Transcriptomics Reveals the ERF2-bHLH2-CML5 Module Responses to H_2_S and ROS in Postharvest Calcium Deficiency Apples. Int. J. Mol. Sci..

[15] Hu K., Peng X., Yao G., Zhou Z., Yang F., Li W., Zhao Y., Li Y., Han Z., Chen X. (2021). Roles of a Cysteine Desulfhydrase LCD1 in Regulating Leaf Senescence in Tomato. Int. J. Mol. Sci..

[16] Li H., Chen H., Chen L., Wang C. (2022). The Role of Hydrogen Sulfide in Plant Roots during Development and in Response to Abiotic Stress. Int. J. Mol. Sci..

